# Predicting Soil Properties and Interpreting Vis-NIR Models from across Continental United States

**DOI:** 10.3390/s22093187

**Published:** 2022-04-21

**Authors:** Christopher M. Clingensmith, Sabine Grunwald

**Affiliations:** Soil and Water Sciences Department, University of Florida, 2181 McCarty Hall, P.O. Box 110290, Gainesville, FL 32611, USA; sabgru@ufl.edu

**Keywords:** soil spectroscopy, visible-near-infrared, soil carbon, soil nutrients, soil texture, partial least squares, machine learning, continental United States

## Abstract

The United States NRCS has a soil database that has data collected from across the country over the last several decades. This also includes soil spectral scans. This data is available, but it may not be used to its full potential. For this study, pedon, horizon and spectral data was extracted from the database for samples collected from 2011 to 2015. Only sites that had been fully described and horizons that had been analyzed for the full suite of desired properties were used. This resulted in over 14,000 samples that were used for modeling and eight soil properties: soil organic carbon (SOC); total nitrogen (TN); total sulfur (TS); clay; sand; exchangeable calcium (Ca_ex_); cation exchange capacity (CEC); and pH. Four chemometric methods were employed for soil property prediction: partial least squares (PLSR); Random Forest (RF); Cubist; and multivariable adaptive regression splines (MARS). The dataset was sufficiently large that only random subsetting was used to create calibration (70%) and validation (30%) sets. SOC, TN, and TS had the strongest prediction results, with an R^2^ value of over 0.9. Ca_ex_, CEC and pH were predicted moderately well. Clay and sand models had slightly lower performance. Of the four methods, Cubist produced the strongest models, while PLSR produced the weakest. This may be due to the complex relationships between soil properties and spectra that PLSR could not capture. The only drawback of Cubist is the difficult interpretability of variable importance. Future research should include the use of environmental variables to improve prediction results. Future work may also avoid the use of PLSR when dealing with large datasets that cover large areas and have high degrees of variability.

## 1. Introduction

The chemometric modeling of soil properties using diffuse reflectance spectroscopy (DRS) spans over two decades. The attractive feature of soil DRS is that it is a rapid, cheap and non-destructive method that does not require reagents or produce chemical waste relative to traditional laboratory soil analysis, so it has been promoted as a complimentary surrogate to traditional analysis especially for large research projects [1,2,3,4,5]. Most early studies focused on small-scale data sets, in terms of the number of samples acquired and/or the geographic extent of the study location, e.g., [6,7,8,9,10,11,12]. These studies typically modeled total carbon (TC), soil organic carbon (SOC) or organic matter (OM), inorganic carbon (IC), total nitrogen (TN), moisture content, sand, clay, pH and/or electrical conductivity (EC). Some studies had also modeled other properties specific to their objectives, such as total elemental content e.g., [13], extractable nutrients e.g., [14], biological properties e.g., [15] or engineering properties e.g., [16]. Some studies were focused on underdeveloped or under-studied regions to test the capability of soil chemometric modeling and determine if the technology would be beneficial [17,18,19,20,21,22,23,24,25,26]. Other studies have tested the efficacy of model transfer [27]. As progress was made and the value of DRS was realized (as well as increases in computing power), larger projects came about, aimed at collecting and scanning thousands of samples over a country or continent to build large spectral datasets.

For the purposes of this paper, we are defining a large spectral library (LSL) as one with 1000 or more samples. For simplicity, we are only reporting one paper per study, since some authors have multiple papers published around the same time using the same dataset but with different goals. A review of the literature showed that the earliest documented LSL soil chemometric study was by Shepherd and Walsh [24], where several soil properties from over 1000 samples were modeled using multivariate adaptive regression splines (MARS). The strongest models were derived for exchangeable Ca, exchangeable Mg, effective cation exchange capacity (CEC) and SOC where R^2^ value greater than 0.9 were achieved. Brown et al. [28] used over 4000 compositionally diverse samples from a global sample set to model soil properties using boosted regression trees to predict SOC, CEC and clay with good results. Gogé et al. [29] used over 2000 French national samples to produce strong model predictions for SOC, CEC and clay and good predictions for sand. The same year the results of modeling several soil properties across Australia, including SOC, TN, clay and sand, were reported with moderate results [30]. Several LSL studies were published between 2013 and 2020, providing the results on one-to-three soil properties, most commonly SOC (or SOM), clay, sand and TN [31,32,33,34,35,36,37,38,39]. More recent attempts have use LSLs with over 10,000 samples for soil property modeling and prediction. These include the LUCAS dataset, that covers most of Europe [40,41], the Brazilian Soil Spectral Library (BSSL) that covers all of Brazil [42] and global soil datasets [43].

In the US, the Natural Resource Conservation Service’s (NRSC) Kellogg Soil Survey Laboratory (KSSL), under the United States Department of Agriculture (USDA), has been agency that collects, measures and records data from soil pedon and horizon observations across the United States. This data has been entered into a large database spanning several decades of work. More recently, the NRCS-KSSL has been scanning soils in the Vis-NIR and NIR regions using diffuse reflectance spectroscopy instrumentation, adding this data to the database. Despite the availability of both Vis-NIR and MIR data, MIR spectra have been the primary dataset of interest to researchers lately e.g., [44,45,46,47,48]. The use of the Vis-NIR data from the NRCS-KSSL spectral library has not been published too considerably [45,49,50]. This discrepancy may be due to the presumption that chemometric models built from MIR data greatly outperform Vis-NIR models, since the MIR region contains the fundamental vibrations from absorption. Bellon-Maurel and McBratney [4] reviewed the applications of both NIR and MIR on different forms of soil C, finding small improvements using MIR over NIR. These improvements are greater when the calibration and validation sets are very similar to each other. Despite this, there are several instances where Vis-NIR or NIR outperforms MIR when predicting soil nutrients or fertility parameters [51,52,53,54,55,56], and one does not know how data will perform until it has been used to model.

The use of Vis-NIR for the prediction of soil properties still warrants study, especially when considering instrument environmental sensitivity, instrument costs and labor of MIR measurements. Additionally, the data in the NRCS-KSSL database has not been fully utilized for soil spectral modeling, so different approaches and goals are still needed. For US stakeholders, this is critically important. US soils are highly diverse, spanning different parent materials, climates, ecologies, land uses, topography, etc. [57], and VNIR modeling across large regions is still of major interest to address soil quality, soil health and soil quality [58,59]. The goal of this study was to model and predict soil properties using chemometric and machine learning methods that allow for spectral interpretation. The objectives of this study were to (1) apply chemometric and machine learning methods to predict soil properties using Vis-NIR spectra and soil pedological and analytical data from the NRCS-KSSL spectral library, (2) compare model performance across modeling approaches and (3) assess and compare wavelength importance between approaches and attribute important wavelengths to functional groups.

## 2. Materials and Methods

### 2.1. Dataset Description and Extraction

The data used in the study was obtained from the Kellogg Soil Science Laboratory (KSSL) through the Natural Resource Conservation Service (NRCS) circa June 2016. This data is of high quality due to the standardized and well-documented soil sample collection, preparation and analytical methods employed by the NRCS [60]. The database contains pedon site, soil layer, measured soil properties and Vis-NIR soil reflectance data. Soil properties were measured using widely accepted laboratory analytical methods. The Vis-NIR data was collected on a visible and near-infrared spectrometer (ASD LabSpec 2500, Analytical Spectral Devices, Boulder, CO, USA) in the spectral range of 350–2500 nm with spectral resolution of 1 nm. The soil sample (ground < 2 mm fraction) was placed in sample holder with a quartz glass window and pressed. The sample was then placed onto the Muglamp. The sample was illuminated from the bottom of the sample holder and scanned 100 times; the scans were then averaged into a single spectrum.

The KSSL data was stored in a Microsoft Access database, which was queried to extract soil pedon data, soil layer (horizon) data. The Vis-NIR spectra was held in a separate folder where the filenames are listed in the database. The query was specified so that soil layer data was only returned if it met the following conditions: it had a soil horizon designation, soil layer top and bottom depths were recorded, soil profile latitude and longitude was measured down to seconds, it was classified at the soil taxonomic order level, had an existing Vis-NIR spectra file, and was sampled from within the contiguous United States. The query was performed for each soil property of interest, which included: soil organic carbon (SOC); total nitrogen (TN); total sulfur (TS); total clay; sand; exchangeable calcium (Ca); cation exchange capacity (CEC); and pH (1:2 0.01M CaCl_2_). The filename of the spectrum for each layer was then used to compile the spectra for all the extracted layers into a single file.

The soil properties were measured according to methods in the Kellogg Soil Survey Laboratory Methods Manual [60]. The percentage of SOC was calculated from the difference of percent total carbon by combustion (Procedure Code: 4H2a) and calcium carbonate equivalent by hydrochloric acid dissolution with manometer (Procedure Code: 4E1a1a1a-2) after conversion to percent inorganic carbon. The percentage of TN and TS were measured by combustion (Procedure Code: 4H2a). The clay faction was measured using the Pipette method and the sand faction via sieving (Procedure Code: 3A1a1a). CEC and Ca_ex_ were determined using ammonium acetate with KCl displacement (Procedure Code: 4B1a2a). Soil pH was measured with 1:2 0.01 M CaCl_2_ suspension (Procedure Code: 4C1a2a2).

### 2.2. Data Screening and Processing

The extracted data was split into pedon (profile/site) data and layer (horizon) data. Pedon data, with coordinates, was imported into ArcMap to check the quality of the coordinate data. Several points were clearly not correct, and the best attempts to correct the data were made based on auxiliary data (project identification number, state, etc.) and recognizing common translation errors of numbers from paper records. Points that could not be reconciled were removed. The remaining points were used to create the final pedon data table. Layer/horizon data, which contains horizon designations and measured soil property data, was screened to ensure values make sense, and did not exceed theoretical limits. For example, the three components of soil texture (clay, silt and sand) were summed to ensure they do not exceed 100 percent total. No records were flagged and removed as erroneous data, but some assumptions were made due to the nature of some methods. For example, inorganic carbon was measured using hydrochloric acid dissolution and manometer which occasionally resulted in negative values. In these cases, it was assumed that there was no carbonate, and the value was changed to zero. Some horizons did not have a measurement for every soil property but that was due to the type of horizon it was. For example, some O horizons did not have any measurements for soil texture.

The spectral data was screened to ensure reflectance measurements did not exceed theoretical limits, from 0.0 to 1.0. A total of 68 records were flagged for removal: 15 for containing positive or negative infinity values, 50 for containing ‘NA’ values and three for containing all 1.0 values. Principal component analysis and Mahalanobis distances confirmed that these samples were outliers, and they were subsequently removed. The remaining spectra were processed to remove an artifact cause by the detectors in the spectrometer. A Savitzky-Golay cubic smoothing filter with a window size of nine was applied to the spectral data to reduce noise and enhance spectral features [61]. The spectral range of the data was reduced to 400–2475 nm to remove potential noisy detector edge effects. The final pedon, layer and spectral data were matched by the layer identifier to create the final datasets for analysis.

### 2.3. Chemometric Modeling

The soil spectra were transformed using several pretreatment methods prior to chemometric modeling, as the best treatment was not known a priori. These techniques included Savitzky-Golay smoothing (SG-S), Savitzky-Golay first and second derivatives (abbreviated as SG-1d and SG-2d, respectively), standard normal variate (SNV), log transformation (LOG) and continuum removal by subtraction and division (CR-S and CR-D, respectively).

Data screening resulted 14,574 samples/layers for modeling. Due to the size of the dataset, random sampling was determined to be an adequate method for splitting the samples into calibration and validation sets (70% and 30%, respectively). The calibration and validation sets were the same for all soil properties modeled. Four methods were used for chemometric modeling. Partial least squares regression (PLSR) has been a stable method in chemometrics. Here, PLSR was performed with the R-package ‘pls’ using the orthogonal scores algorithm [62]. Cross-validation was used with 10-folds, and the maximum number of latent variables was set at 200. The optimal number of latent variables was selected based on the minimized root mean squared error (RMSE). The tree-based method Random Forest was used as a non-parametric, data mining method [63]. The R-package ‘ranger’ was used to create the Random Forest models with the number of trees set to 3625 (approximately 25% of the number of samples). The Cubist method, a regression rules algorithm that combines a tree-based structure with linear regression models, was incorporated due to its ability to handle large datasets with high dimensionality [64]. Cubist was employed with R-package ‘Cubist’ with 50 committee and 0 or 9 neighbors. The fourth modeling method was multivariate adaptive regression splines (MARS), a method designed for high-dimensional data that employs splines that are able to capture multi-level interactions [65]. MARS models were created with the R-package ‘earth’ with the maximum number of degrees set to 10 and the maximum number of terms set to 50. Ten-fold cross-validation for Cubist and MARS was performed with the R-package ‘caret’ using the minimization of RMSE for model parameter or term selection.

## 3. Results and Discussion

### 3.1. Data Summary and Statistics

**Figure 1:**
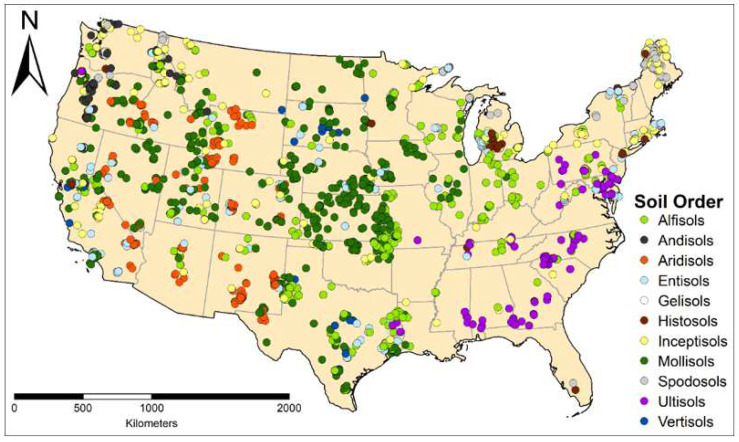
Map of soil pedons used in this study across the continental United States.

Data that met the criteria mentioned in the previous section resulted in 2618 pedons and 14,428 horizons/layers. These soils and the resulting data were collected, described and measured between 2011 and 2015, and were sampled within the continental United States. Table 1 shows the number of pedons represented by each soil order according to the USDA soil taxonomy [66]. The pedon data is dominated by Molisols, which represent 32% of the pedons, followed by Alfisols (16%) and Inceptisols (12.3%). The dataset does not well represent Andisols, Gelisols, Histosols and Vertisols, of which there are less than 100 pedons of each, and Oxisols, where there are none. Figure 1 shows the distribution of the pedons across the continental United States. Since this data was extracted after it has been collected, the distribution of pedon points reflects the projects that the NRCS was working on between 2011 and 2015, and thus are not evenly distributed across the United States. The map shows a large concentration of points around Kansas, with points extending into Nebraska, Oklahoma, Arkansas and Iowa, where much farmland is found. However, parts the southeastern US are not well represented, including much of Florida and the southern Atlantic Seaboard. Parts of New England, Kentucky, Missouri, Arizona and Montana are also not well represented.

Table 2 shows the distribution of the soil layer data within horizon designations, according to the 12th Edition of the Keys of Soil Taxonomy [66]. Combinational and transitional horizon designations were split into master horizon and subordinate horizon regardless of the type, so an AB horizon and an A/B horizon would both have an A master horizon and a B subordinate horizon. B master horizons are the most dominant, which is likely because many soil profiles tend to have more than one B horizon. O and E master horizons are less common because they require certain conditions to form. L and R master horizons are not well represented in the dataset, but they were not removed since stratifying by horizon designation is not the focus of this study. The horizon suffixes t, k, p, g and w are the most commonly occurring in this dataset (not shown).

Table 3 provides summary statistics of selected soil properties. The table also includes the number of instances where a layer had a no value (NA) or a value of 0 recorded. In this dataset, SOC ranges from 0 to 57.2% with a mean of 2.5% and median of 0.52%, thus skewness and kurtosis are high. There is one record with a ‘NA’ value and 288 records with a value of 0%. TN has a range of 0 to 4.17%; the mean is 0.17% and the median is 0.07%, which results in a high positive skewness and very high kurtosis. There are 10 layers with values of ‘NA’ and 1156 layers with a value of zero. TS is highly skewed and peaked. It ranges from 0 to 25.2% but has very small mean and median of 0.17% and 0.01%, respectively. TS also has 10 layers with a value of ‘NA’ and 4562 layers with a value of zero. The range of total clay extends from 0 to 96.1%, with a mean of 21.4% and median of 19.6%. Total sand has a range of 0.20 to 100%, with a mean of 42.6% and a median of 38.7%. Both properties are not very skewed, and there are 341 layers with a value of ‘NA’. Exchangeable Ca of these soils ranges from 0 to 372 cmol_+_ kg^−1^ with a mean of 22.7 cmol_+_ kg^−1^ and median of 10.5 cmol_+_ kg^−1^, and is positively skewed and very peaked. CEC has a range of 0 to 585 cmol_+_ kg^−1^; the mean is 17.3 cmol_+_ kg^−1^ and median is 13.3 cmol_+_ kg^−1^. The distribution of CEC is positively skewed and highly peaked. Ca_ex_ and CEC both have 146 layers with values of ‘NA’. Measured pH in 1:2 0.01M CaCl_2_ ranges from 2.3 to 10.5 with a mean of 6.0 and a median of 5.8. There are 173 layers with a value of ‘NA’. The records with ‘NA’ values were not analyzed with the specific method, as it was not appropriate for the type of soil horizon.

### 3.2. Prediction Results

The prediction results of all four modeling methods are presented in Table 4. A cursory glance shows that the metrics for validation may be slight better than those of cross-validation with TS models displaying this behavior to the largest degree. This may be because one random subsetting into calibration and validation sets was performed, so that each model was constructed from data from the same samples regardless of modeled property. This may have caused the data to be unbalanced in some respects and cause validation metrics to be better than calibration/cross-validation. However, each model was constructed using 10-fold cross-validation without resampling, which is the standard technique to combat overfitting. Stratified cross-validation is one technique that could be used to help, but it was not part of the scope of this paper.

Overall, model performances on the validation set for each soil property do not vary widely except for clay and sand. SOC, TN and TS models were strong in general with validation R^2^ values greater than 0.9 for these properties. RPD values are high (>2.4) but RPIQ values are very low, mostly less than 1.0. The low RPIQ values are likely low due to the high degree of skewness and kurtosis present in these data (Table 3). Guerrero et al. [32] had similar results modeling SOC using PLSR with R^2^ value of 0.91 using samples taken throughout Spain. There were able to achieve a lower RMSE (0.99%) but this likely due to the smaller area of the study compared to the continental US. Liu et al. [35] used Cubist to achieve R^2^ value of 0.94 and RMSE of 0.37% modeling SOC. They were able to achieve lower RMSE since the study was focused on forest soils in China. Another paper that also used samples from the NRCS-KSSL library (not necessarily the same samples as this study) had R^2^ values of 0.49 and 0.66 for PLSR and Cubist, respectively, which is much lower than this study despite having used a much more pruned dataset (e.g., no SOC values > 10%) [45]. The model that they were promoting was also had poorer performance than our Cubist model using Vis-NIR spectra.

Only a few other large library studies have modeled TN, however none of them were able to reach the results obtained in this study. Viscarra Rossel and Webster [30] modeled log-transformed TN using Cubist, and reported an RPD values of 2.11, while this study achieved a value of 3.32 using Cubist. Another study achieved an R^2^ value of 0.88, despite a very low RMSE (0.07%) and high RPD (3.69), however this study was conducted in a small geographic area, which is likely why those metrics are good [33]. Yang et al. [41] reported poor performance using PLSR with R^2^ of 0.57 in Europe using the Land Use and Coverage Area Frame Survey (LUCAS) data set. This is a comprehensive soil dataset that covers the European Union see [67]. None of the papers reviewed included TS as a soil property to be modeled, so our study is the first to do so at the continental/national scale. Considering the very high skewness and kurtosis of the TS data and the number of ‘0′ values (>4500), our models performed very well with little to no bias, and high RPD values (2.79–5.28).

Models predicting clay and sand were fair to good. Clay model R^2^ values ranged from 0.65 to 0.85. RPD values ranged from 1.75 to 2.53 and RPIQ values were higher than the previous models from 2.58 to 3.78. The Cubist RMSE was lowest at 6.35%. Brown et al. [28] used samples from an earlier version of the KSSL-NRCS library, and was able to model clay with better results using bagged regression trees (R^2^: 0.91; RMSE: 5.4%). Their study only used one sample from each pedon. The modeling of clay across France was also successful yielding R^2^ of 0.90, RMSE of 3.03% and RPD of 4.39 [29]. These results are likely, since only surface samples (0–30 cm) were used in that paper, while ours used multiple horizons to depths greater than 1 m. Clay and sand was also modeled by Ng et al. [45], and again the prediction metrics of PLSR and Cubist were much lower than what we obtained, including RPIQ values that were closer to 1.0.

Sand model R^2^ values were lower compared to the clay models from 0.57 to 0.75. The RMSE values were high at roughly 15–19%. This resulted in RPD values of less than 2.0, except for the Cubist model that was just above 2.0, however the RPIQ values were higher. Viscarra Rossel and Webster [30] modeled sand across most of Australia, and were also able to predict sand with slightly better success with Cubist (RMSE: 12.0%; RPD: 2.06). The study also used soil samples from multi-layer pedons, and this could be the reason for the similar results. Modeling sand in Brazil was met with more success where the R^2^ was 0.96 and RMSE was 13.8%, though the study area was small, only covering several municipalities within Sao Paulo State, so the geographic variability [34]. Ogen et al. [37] used spectral analysis and clustering coupled with PLSR in Israel, and obtained higher R^2^ and lower RMSE values than our PLSR model, but our Cubist model produced better results.

Models predicting chemical properties (Ca_ex_, CEC, pH) were good overall. There are few papers in the literature where Ca_ex_ is modeled from large libraries. Ca_ex_ models in this study had little variation in terms of prediction metric values. R^2^ values ranged from 0.82 to 0.90 with high RPD values (2.19–3.27). Shepard and Walsh [24] had greater success modeling Ca_ex_ with MARS with R^2^ of 0.88 and RMSE of 2.8 cmol_+_ kg^−1^, but the data was only from tropical topsoil samples of eastern and southern Africa. Ca_ex_ was also predicted across Australia using Cubist with RMSE of 3.77 cmol_+_ kg^−1^ and RPD of 2.34. The RMSE is about 3–4 times lower than our Cubist model, but that is likely a function of the smaller range and lower skewness of the data [30].

CEC modeling has been slightly more prevalent in the literature. The predictive success of our CEC models was moderate with R^2^ values 0.70–0.86 and acceptable RPD values. RPIQ values were lower than RPD values. CEC was modeled in Africa (as an effective CEC) with slightly better results (R^2^: 0.88; RMSE: 3.3 cmol_+_ kg^−1^), but again, these were only topsoil samples [24]. Brown et al. [28] also modeled CEC with an older version of the NRCS-KSSL database producing similar results. Ng et al. [45], using a more recent version of the database, produced poorer results using PLSR and Cubist with R^2^ values 0.61 and 0.68, respectively, even though the data used was highly constrained.

The pH_Ca_ model metrics were moderate to good with Cubist producing the best results (R^2^: 0.87; RMSE: 0.52; RPD: 2.75). Few other papers have presented modeling results of pH_Ca_ using large spectral libraries. Viscarra Rossel and Webster [30] predictions of pH_Ca_ across Australia with Cubist resulted in similar results as this study (RMSE: 0.57; RPD: 2.16). The LUCAS dataset across Europe also produced similar results with PLSR with R^2^ of 0.81 and RMSE of 0.59 [41].

### 3.3. Model Comparisons

The spectral pretreatments that produced the strongest models shows patterns across the metrics table (Table 4). All Random Forest models performed best with the Savitzky-Golay 1st derivative (SG-1d). Cubist models best performed with continuum removal, by substitution (CR-S) or division (CR-D) and standard normal variate (SNV) treatments. MARS models mostly performed best with SG-1d as well, but there were two instances of CR-D. PLSR models had a variety of pretreatments, which produced the strongest models.

**Figure 2:**
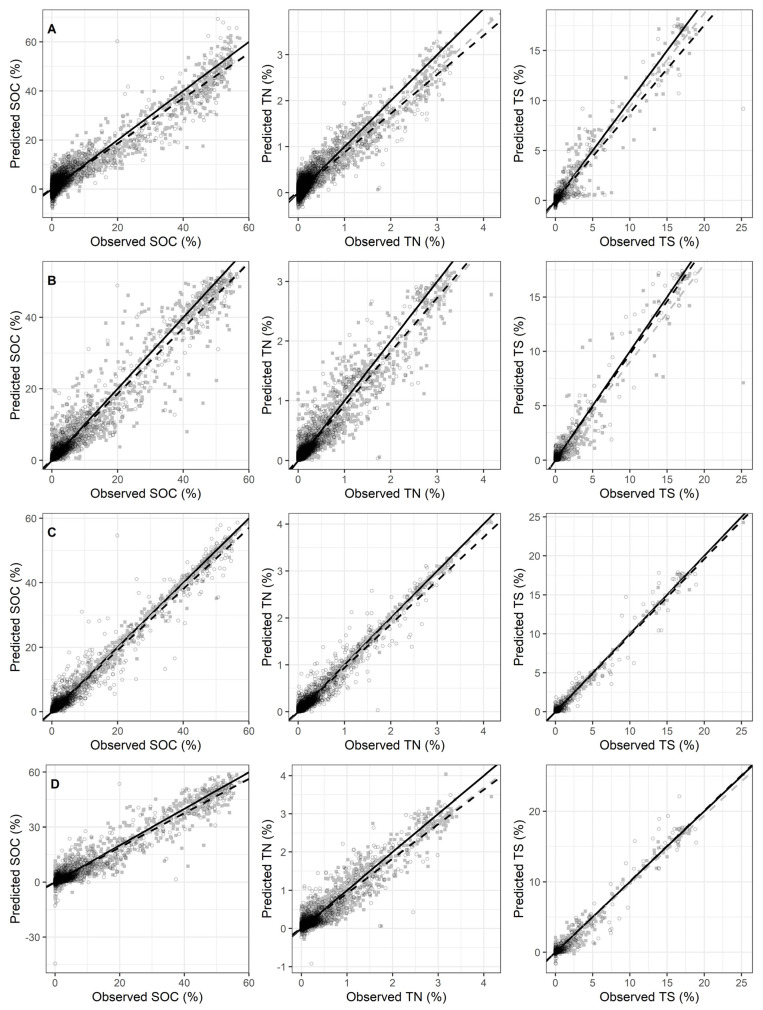

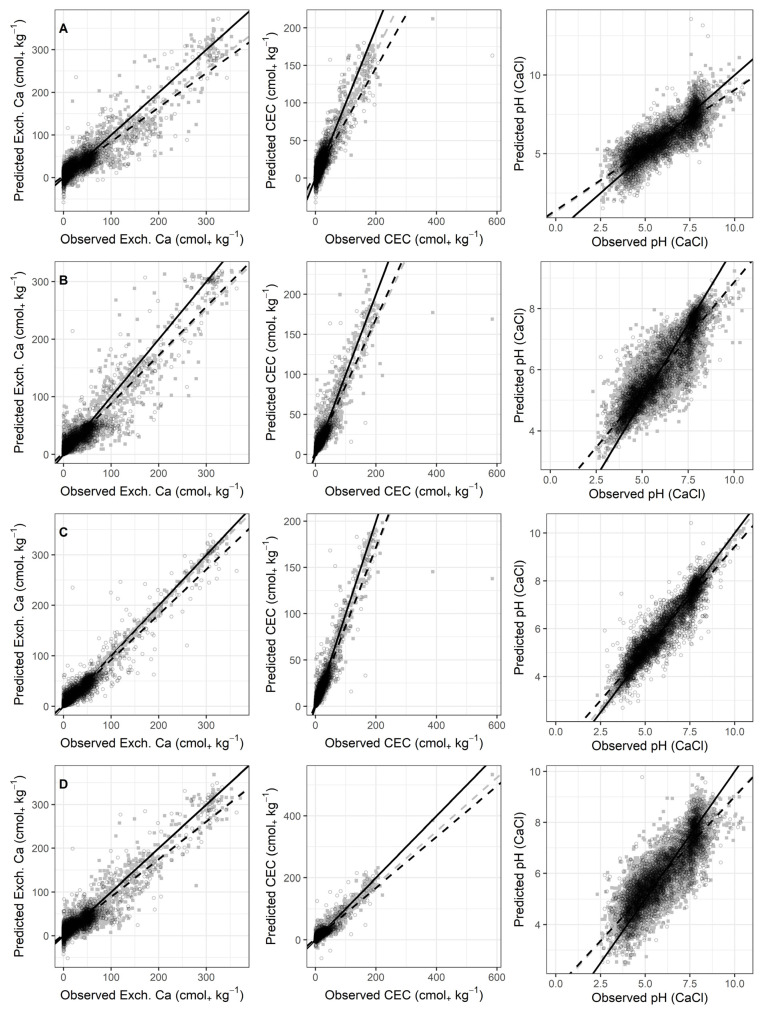

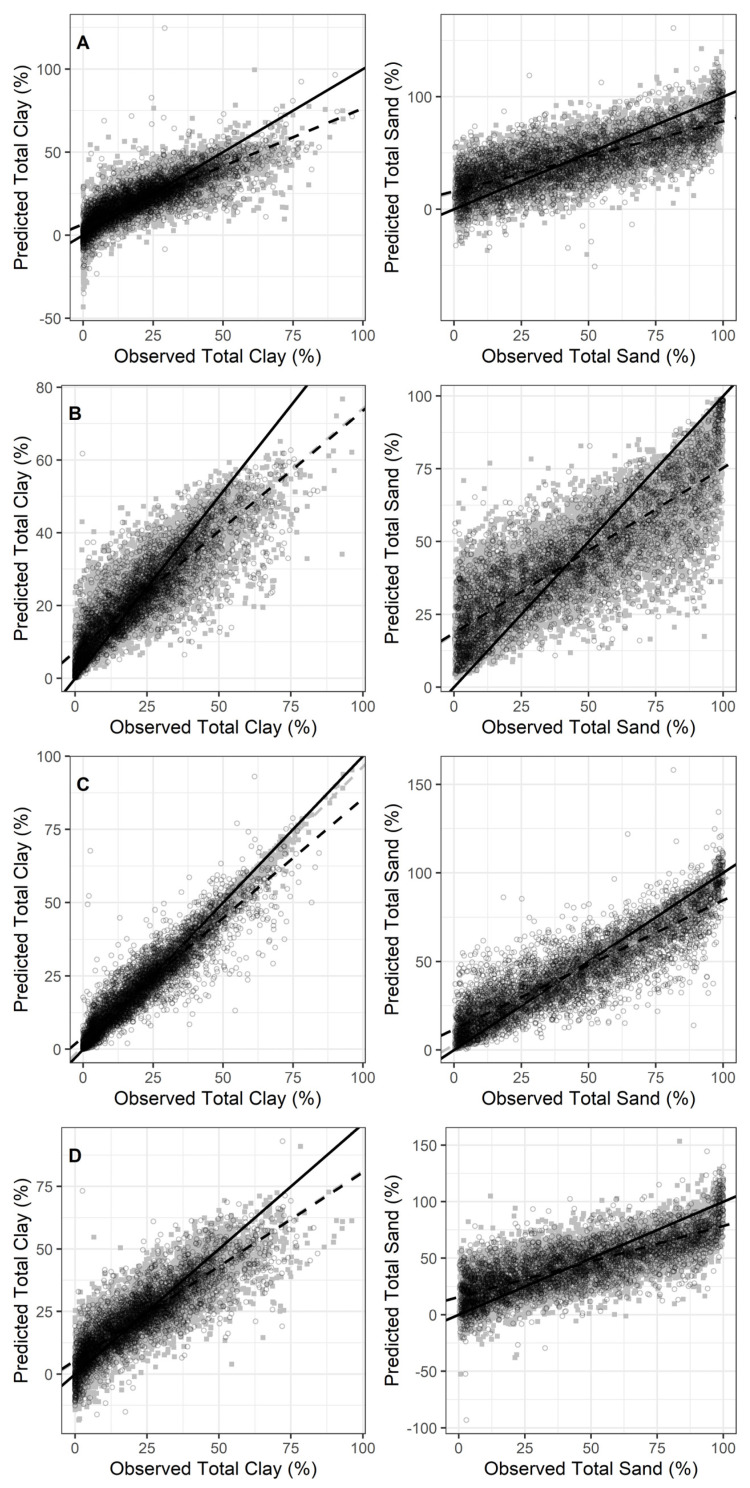
Observed versus predicted values for calibration and validation sets. Calibration values are shown with grey filled squares (■) and validation values are shown with (black open circles (○). Model types are organized by rows: PLSR models in row (**A**), RandomForest models in row (**B**), Cubist models in row (**C**) and MARS models in row (**D**).

PLSR produced the worst predictions overall, followed by MARS. The main limitation of PLSR and MARS is their ability to extrapolate, which allows extreme values outside the potential domain or far from the observed values to be predicted. This is apparent in the SOC validation predictions from PLSR and MARS, the TN validation predictions from MARS, the clay validation predictions from PLSR and MARS and the sand validation predictions from PLSR and MARS. The PLSR predictions of SOC in Figure 2 show that there are two populations of points. The first population appears to be from 0% to 40% and the second population is 40% to 60%, with a kink at 40%, which coincides with greater underprediction in the 30% to 50% range. Despite this, PLSR still predicted SOC moderately well. This graphic is in stark contrast to the PLSR SOC prediction graphic presented in Ng et al. [45], where the results suffer from severe underprediction, which increases as SOC increases even when using the same database. The same pattern may be present in the PLSR TN results (Figure 2) where a kink appears between 2.0% and 2.5%. This is not surprising, considering that SOC and TN are highly correlated with each other. This is additionally supported by the results of pH models shown in Figure 2. Here, there appears to be a second population starting around 7.5, where the observed and predicted points lie in a more vertical line than with the overall trend. The MARS models have a few instances of extreme negative value predictions, namely SOC, TN and sand (Figure 2).

Overall, the Cubist method produced the strongest models for each soil property. Figure 2 shows the observed versus predicted graphs for the calibration and validation sets. The graph shows the Cubist models (row C) have the predicted values closest to the 1:1 line. This is also true for properties that tend to have a cloud around the trend line, like clay and sand. There are three instances of extreme predictions made by Cubist in validation of SOC, Ca_ex_ and clay. This contrasts with the other methods that have more spread around the 1:1 line. The RF models appear to have a larger amount of spread despite having the second-best prediction results. The benefit of machine learning methods like Cubist and RF is that they usually do not make predictions outside the domain of the input response data, e.g., negative values. There is one instance where RF predicted outside the potential domain with the sand validation predictions.

### 3.4. Model Interpretations

Figure 2 shows the importance of each wavelength for each soil property and modeling method. The PLSR graphic (Figure 2) portrays the variable importance in the projection (VIP) derived from the method by Mevik [68], and is defined as importance if the VIP value is greater than one. The wavelength importance from the remaining methods was calculated using the R package caret [69]. The PLSR VIP plots highlight the diagnostic regions that are commonly associated with soil spectroscopy. These include: the visible region (400–700 nm), which is associated with organic matter, iron oxides and other chromophores; ~1400–1450 nm, associated with overtones of hydroxyl group (-OH) fundamental vibrations; ~1900–1950 nm, associated with combinations of water and -OH vibrations; ~2200–2300 nm, associated with combinations of metal-OH and -OH vibration; and ~2300–2400 nm, associated with combination vibrations from C-H bonds [70]. Interestingly, the fundamental vibrations from C-H bonds at 1700 nm is not present in any of the plots, including SOC. However, the features near the 2300–2400 nm region may be from -OH and carbonate absorption [70]. The SOC, TN and CEC plots show that the red region is important for model predictions, so there may be an association with iron oxides. The most likely sources are Bh, Bs and Bsh horizons, where organic matter and sesquioxides are found together. The reason the pH plot appears much different than the other plots is due to the use of the SG-2d transformed spectra.

The RF plots (Figure 2) appear much different than the PLSR due to each model using the SG-1d transformed spectra. Since the spectra are the first derivative, the RF models should have targeted features derived from increases or decreases in reflectance with increasing wavelength. Most soil properties, except TS, have small peaks in the visible region. CEC and TN appear to relate to the violet-blue region, and the models may have been training from the same factors despite being not highly correlated with each other. The SOC model relies most on the ~2150–2200 nm region, likely associated with water and -OH vibrations around 2200–2300 nm. This region was also important for CEC, pH and TN, showing a very similar pattern to the SOC importance graph. Interestingly, the C-H combination vibrations near 2400 nm do not contribute to the SOC model. However, Ca_ex_ and pH have a small importance peak near this region, and is most likely associated with -OH and carbonate absorption features. Ca_ex_ also has a major peak near 1900–1950 nm, which may be due to strong absorption features of hydrated Ca-sulfates [71]. There are two semi-major peaks in the 1700–1800 nm region, which may be associated with C-O vibrations in carbonates [72]. There is also a semi-major peak near 2000 nm. Clay has a major and semi-major peak in the 1800–1850 nm region and a semi-major peak near 2300 nm which are shifted from the water absorption features at 1900 and 2200 nm, which are commonly associated with clay minerals [72]. Sand has a major peak near 2250 nm and semi-major peaks near 1900 and 2200 nm. In this case, the models may be selecting the clay features to build inverse relationships to derive sand content, and this may also be why sand models tend to underperform relative to clay minerals. TS bears a much different pattern showing a major peak near 1775 nm, semi-major peaks near 1450 nm and 1750 nm and several smaller peaks in between. The peak at 1450 nm may be from a strong absorption feature of hydrated Ca-sulfates [72].

The importance graphs for the Cubist and MARS models are much more complicated than the plots for PLSR and MARS. The Cubist method incorporated nearly all wavelengths into the models, so there are not well-defined peaks. Nearly each soil property modeled has a sharp peak in the 1850–1950 nm region (-OH absorption). Ca_ex_ has additional sharp peaks near 2200 and 2350 nm, the former of which is likely due to clay minerals. CEC has sharp peaks at 700 and 2200–2500 nm. Clay has a broad peak near 750 nm and two sharp peaks between 1300 and 1400 nm. The pH graph is more difficult to interpret, as there are not any significant sharp peaks other than the one near 1850 nm. Sand has a peak near 760 nm and two sharp peaks near 1350 and 1500 nm. SOC has a small but sharp peak near 600 nm and a sharp peak near 2250 nm. TN also has sharp peaks near 600 and 2250 nm, as well as sharp peaks near 1400 and 1700 nm. Unfortunately, these are not easily assignable to known N-H bend or stretch overtone features [73], but the 1700 and 2250 nm features are close to bands determined to be sensitive to soil TN through use of an evolutionary algorithm [74]. Models for TN may be drawing on the close correlation of TN with SOC, rather than the absorption features of N-H bonds, as the concentration of TN is much smaller relative to SOC. TS has a small sharp peak near 750 nm and taller peaks neat 1400 and 1750 nm. The important wavelengths selected by the MARS models generally span broader regions than the importance peaks of the other models, but are focused on specific wavelengths. The main regions are 400–800 nm, 1300–1500 nm and 2050–2400 nm, corresponding to the visible region, -OH group fundamental overtones and the collective region of combination vibrations from water/-OH and metal-OH/-OH. SOC, TN and TS are the only properties where a single wavelength was central to the model structure. These wavelengths are near 2200, 2225 and 2400 nm for SOC, TN and TS, respectively.

## 4. Conclusions

The study shows that DRS has the potential to replace traditional soil analyses for samples collected by the NRCS that span a range of soil types, textures, parent materials and climates, and that includes subsurface horizons. Additionally, it utilizes data that has not been fully operationalized in the United States. Four non-black box chemometric methods were explored that produced strong to moderate results. Of the four methods, Cubist models consistently produced the best predictions. Surprisingly, PLSR greatly underperformed compared to Cubist and RF despite being a staple of soil spectroscopy. The dataset may have been too complex for PLSR without a way for the method to establish non-linear relationships. Cubist does use linear regression equations, but it also includes a tree-based structure with conditional nodes; this is what allows it to establish linear and non-linear relationships. The only downside of Cubist is the difficulty of interpreting variable importance because many of the variables are used to a significant degree. Despite this, this study may indicate that PLSR should not be used for large, complex datasets. Complex datasets such as this one will require methods that can account for linear and non-linear relationships, as both of those exist between the soil properties and spectra.

Future work will build upon this research with the addition of spatial environmental variables and the incorporation of stratification into the modeling framework. This research was published separately, as we felt that it would make the paper too long if the results were published together.

## Figures and Tables

**Table 1:** Distribution of soil pedons, extracted from the NRCS-KSSL database, across USDA soil orders and the number of suborders represented for each soil order.

Soil Order	n	%	Suborders Represented
Alfisols	418	16.0	5/5
Andisols	90	3.4	6/8
Aridisols	202	7.7	7/7
Entisols	236	9.0	6/6
Gelisols	25	1.0	3/3
Histosols	76	2.9	5/5
Inceptisols	323	12.3	7/7
Mollisols	838	32.0	6/8
Oxisols	0	0.0	0/5
Spodosols	174	6.7	4/5
Ultisols	194	7.4	3/5
Vertisols	42	1.6	5/6
Total	2618	100	57/70

**Table 2:** Distribution of soil layers, extracted from the NRCS-KSSL database, across horizon designations.

Horizon Designation	Dominant Horizon	Subordinate Horizon
O	564	40
L	6	-
A	3695	193
E	551	297
B	7281	379
C	2328	807
R	3	1

**Table 3:** Summary statistics of selected soil properties from the KSSL dataset and their respective NRCS Procedure Code.

Soil Properties Procedure Codes	Min.	Q1	Mean	Median	Q3	Max.	Range	IQR	SD	CV	Skew.	Kurt.	NAs	Zeros
Soil Organic C (%) Codes: 4H2a and 4E1a1a1a1	0.00	0.18	2.50	0.52	1.46	57.2	57.2	1.28	7.42	2.97	5.15	30.4	1	288
Total N (%) Code: 4H2a	0.00	0.03	0.16	0.07	0.14	4.17	4.17	0.12	0.35	2.24	5.51	38.3	10	1156
Total S (%) Code: 4H2a	0.00	0.00	0.17	0.01	0.02	25.2	25.2	0.02	1.24	7.18	11.5	147	10	4562
Total Clay (%) Code: 3A1a1a	0.00	7.90	21.4	19.6	31.5	96.1	96.1	23.5	15.9	0.70	0.70	3.20	341	231
Sand (%) Code: 3A1a1a	0.20	16.2	42.6	38.7	66.2	100	99.8	50.0	29.4	0.70	0.30	1.90	341	0
Exch. Ca (cmol_+_ kg^−1^) Code: 4B1a2a	0.00	2.10	22.7	10.5	27.4	372	372	25.3	38.7	1.70	4.50	29.7	146	858
CEC (cmol_+_ kg^−1^) Code: 4B1a2a	0.00	6.40	17.3	13.3	21.8	585	585	15.4	20.6	1.20	5.90	75.3	146	10
pH 1:2 0.01M CaCl_2_ Code: 4C1a2a2	2.30	4.80	6.00	5.80	7.4	10.5	8.20	2.60	1.40	0.20	0.20	1.90	173	0

**Table 4:** Cross-validation and validation prediction metrics of the best models for each soil property.

Property	Method	Pretreat.	N *	CV		Validation				
				R^2^	RMSE	R^2^	Bias	RMSE	RPD	RPIQ
SOC (%)	PLSR	LOG	148	0.88	2.59	0.88	0.03	2.48	2.90	0.53
	RF	SG-1d		0.94	1.78	0.95	0.02	1.68	4.24	0.79
	Cubist	CR-S		0.94	1.82	0.95	0.00	1.62	4.39	0.81
	MARS	SG-1d	26	0.92	2.09	0.91	−0.05	2.10	3.40	0.63
TN (%)	PLSR	LOG	197	0.83	0.145	0.84	0.00	0.14	2.50	0.76
	RF	SG-1d		0.91	0.110	0.89	0.01	0.11	2.98	1.05
	Cubist	CR-D		0.92	0.100	0.91	0.00	0.09	3.32	1.16
	MARS	CR-D	43	0.86	0.135	0.84	0.00	0.13	2.44	0.85
TS (%)	PLSR	SNV	48	0.93	0.310	0.87	0.00	0.37	2.79	0.06
	RF	SG-1d		0.91	0.317	0.96	0.01	0.25	5.13	0.09
	Cubist	CR-D		0.92	0.260	0.96	0.00	0.25	5.28	0.09
	MARS	CR-D	30	0.89	0.348	0.95	0.00	0.31	4.22	0.07
Clay (%)	PLSR	SNV	58	0.65	9.35	0.67	0.17	9.03	1.75	2.58
	RF	SG-1d		0.76	7.80	0.77	0.01	7.97	2.01	3.01
	Cubist	SNV		0.85	6.23	0.84	0.15	6.35	2.53	3.78
	MARS	SG-1d	50	0.73	8.14	0.74	0.10	8.19	1.96	2.93
Sand (%)	PLSR	SG-S	158	0.56	19.5	0.57	−0.14	19.30	1.51	2.54
	RF	SG-1d		0.69	16.3	0.72	0.15	16.61	1.78	3.04
	Cubist	SNV		0.75	14.8	0.75	−0.01	14.68	2.02	3.44
	MARS	SG-1d	50	0.60	18.6	0.61	−0.19	18.42	1.61	2.74
Ca_ex_ (cmol_+_ kg^−1^)	PLSR	CR-D	72	0.82	16.2	0.79	0.03	16.23	2.19	1.51
	RF	SG-1d		0.87	13.2	0.88	0.35	13.79	2.86	1.84
	Cubist	CR-D		0.90	11.4	0.91	0.28	12.08	3.27	2.10
	MARS	SG-1d	45	0.83	15.0	0.86	−0.24	15.00	2.63	1.69
CEC (cmol_+_ kg^−1^)	PLSR	LOG	159	0.74	10.4	0.70	0.00	11.66	1.84	1.30
	RF	SG-1d		0.84	8.71	0.84	0.19	7.38	2.49	2.12
	Cubist	SNV		0.86	7.98	0.86	−0.20	6.92	2.66	2.26
	MARS	SG-1d	50	0.73	11.6	0.76	0.06	9.19	2.00	1.70
pH	PLSR	SG-2d	71	0.74	0.715	0.73	0.00	0.74	1.91	3.55
	RF	SG-1d		0.80	0.633	0.82	0.00	0.62	2.29	4.28
	Cubist	CR-D		0.86	0.524	0.87	0.00	0.52	2.75	5.15
	MARS	SG-2d	50	0.74	0.719	0.75	0.01	0.71	2.01	3.76

* N is the number of latent variables for PLSR or the number of terms for MARS.

## Data Availability

Data derived from the National Cooperative Soil Survey Soil Characterization Database obtained from the Kellogg Soil Survey Laboratory (KSSL) of the Natural Resource Conservation Service (NRCS) under the United States Department of Agriculture (USDA).

## References

[1] Shepherd K.D., Walsh M.G. (2007). Infrared Spectroscopy—Enabling an Evidence-Based Diagnostic Surveillance Approach to Agricultural and Environmental Management in Developing Countries. J. Near Infrared Spectrosc..

[2] Rossel R.V., McBratney A.B. (2008). Diffuse Reflectance Spectroscopy as a Tool for Digital Soil Mapping. Digital Soil Mapping with Limited Data.

[3] Du C., Zhou J. (2009). Evaluation of Soil Fertility Using Infrared Spectroscopy: A Review. Environ. Chem. Lett..

[4] Bellon-Maurel V., McBratney A. (2011). Near-Infrared (NIR) and Mid-Infrared (MIR) Spectroscopic Techniques for Assessing the Amount of Carbon Stock in Soils—Critical Review and Research Perspectives. Soil Biol. Biochem..

[5] Nocita M., Stevens A., van Wesemael B., Aitkenhead M., Bachmann M., Barthès B., Ben Dor E., Brown D.J., Clairotte M., Csorba A. (2015). Soil Spectroscopy: An Alternative to Wet Chemistry for Soil Monitoring. Advances in Agronomy.

[6] Dalal R.C., Henry R.J. (1986). Simultaneous Determination of Moisture, Organic Carbon, and Total Nitrogen by near Infrared Reflectance Spectrophotometry. Soil Sci. Soc. Am. J..

[7] Ben-Dor E., Banin A. (1995). Near-Infrared Analysis as a Rapid Method to Simultaneously Evaluate Several Soil Properties. Soil Sci. Soc. Am. J..

[8] Chang C.-W., Laird D.A., Mausbach M.J., Hurburgh C.R. (2001). Near-Infrared Reflectance Spectroscopy–Principal Components Regression Analyses of Soil Properties. Soil Sci. Soc. Am. J..

[9] Bertrand I., Janik L.J., Holloway R.E., Armstrong R.D., McLaughlin M.J. (2002). The Rapid Assessment of Concentrations and Solid Phase Associations of Macro and Micronutrients in Alkaline Soils by Mid-Infrared Diffuse Reflectance Spectroscopy. Aust. J. Soil Res..

[10] Islam K., Singh B., McBratney A. (2003). Simultaneous Estimation of Several Soil Properties by Ultra-Violet, Visible, and near-Infrared Reflectance Spectroscopy. Aust. J. Soil Res..

[11] Bogrekci I., Lee W.S. (2005). Spectral Soil Signatures and Sensing Phosphorus. Biosyst. Eng..

[12] Brown D.J., Bricklemyer R.S., Miller P.R. (2005). Validation Requirements for Diffuse Reflectance Soil Characterization Models with a Case Study of VNIR Soil C Prediction in Montana. Geoderma.

[13] Reeves J.B., Smith D.B. (2009). The Potential of Mid- and near-Infrared Diffuse Reflectance Spectroscopy for Determining Major- and Trace-Element Concentrations in Soils from a Geochemical Survey of North America. Appl. Geochem..

[14] Vašát R., Kodešová R., Borůvka L., Klement A., Jakšík O., Gholizadeh A. (2014). Consideration of Peak Parameters Derived from Continuum-Removed Spectra to Predict Extractable Nutrients in Soils with Visible and near-Infrared Diffuse Reflectance Spectroscopy (VNIR-DRS). Geoderma.

[15] Bellino A., Colombo C., Iovieno P., Alfani A., Palumbo G., Baldantoni D. (2016). Chemometric Technique Performances in Predicting Forest Soil Chemical and Biological Properties from UV-Vis-NIR Reflectance Spectra with Small, High Dimensional Datasets. Iforest-Biogeosci. For..

[16] Waruru B.K., Shepherd K.D., Ndegwa G.M., Kamoni P.T., Sila A.M. (2014). Rapid Estimation of Soil Engineering Properties Using Diffuse Reflectance near Infrared Spectroscopy. Biosyst. Eng..

[17] Awiti A.O., Walsh M.G., Shepherd K.D., Kinyamario J. (2008). Soil Condition Classification Using Infrared Spectroscopy: A Proposition for Assessment of Soil Condition along a Tropical Forest-Cropland Chronosequence. Geoderma.

[18] Bikindou F.D.A., Gomat H.Y., Deleporte P., Bouillet J.-P., Moukini R., Mbedi Y., Ngouaka E., Brunet D., Sita S., Diazenza J.-B. (2012). Are NIR Spectra Useful for Predicting Site Indices in Sandy Soils under Eucalyptus Stands in Republic of Congo?. For. Ecol. Manag..

[19] Camacho-Tamayo J.H., Rubiano S., Hurtado S., del Pilar M. (2014). Near-Infrared (NIR) Diffuse Reflectance Spectroscopy for the Prediction of Carbon and Nitrogen in an Oxisol. Agron. Colomb..

[20] Clingensmith C.M., Grunwald S., Wani S.P. (2019). Evaluation of Calibration Subsetting and New Chemometric Methods on the Spectral Prediction of Key Soil Properties in a Data-Limited Environment: Evaluation of Subsetting and New Chemometric Methods. Eur. J. Soil Sci..

[21] Cozzolino D., Morón A. (2003). The Potential of Near-Infrared Reflectance Spectroscopy to Analyse Soil Chemical and Physical Characteristics. J. Agric. Sci..

[22] Daniel K.W., Tripathi N.K., Honda K. (2003). Artificial Neural Network Analysis of Laboratory and in Situ Spectra for the Estimation of Macronutrients in Soils of Lop Buri (Thailand). Aust. J. Soil Res..

[23] Nawar S., Buddenbaum H., Hill J., Kozak J., Mouazen A.M. (2016). Estimating the Soil Clay Content and Organic Matter by Means of Different Calibration Methods of Vis-NIR Diffuse Reflectance Spectroscopy. Soil Tillage Res..

[24] Shepherd K.D., Walsh M.G. (2002). Development of Reflectance Spectral Libraries for Characterization of Soil Properties. Soil Sci. Soc. Am. J..

[25] Tziolas N., Tsakiridis N., Ben-Dor E., Theocharis J., Zalidis G. (2019). A Memory-Based Learning Approach Utilizing Combined Spectral Sources and Geographical Proximity for Improved VIS-NIR-SWIR Soil Properties Estimation. Geoderma.

[26] Volkan Bilgili A., van Es H.M., Akbas F., Durak A., Hively W.D. (2010). Visible-near Infrared Reflectance Spectroscopy for Assessment of Soil Properties in a Semi-Arid Area of Turkey. J. Arid Environ..

[27] Grunwald S., Yu C., Xiong X. (2018). Transferability and Scalability of Soil Total Carbon Prediction Models in Florida, USA. Pedosphere.

[28] Brown D.J., Shepherd K.D., Walsh M.G., Dewayne Mays M., Reinsch T.G. (2006). Global Soil Characterization with VNIR Diffuse Reflectance Spectroscopy. Geoderma.

[29] Gogé F., Joffre R., Jolivet C., Ross I., Ranjard L. (2012). Optimization Criteria in Sample Selection Step of Local Regression for Quantitative Analysis of Large Soil NIRS Database. Chemom. Intell. Lab. Syst..

[30] Rossel R.A.V., Webster R. (2012). Predicting Soil Properties from the Australian Soil Visible–near Infrared Spectroscopic Database. Eur. J. Soil Sci..

[31] Castaldi F., Chabrillat S., Chartin C., Genot V., Jones A.R., van Wesemael B. (2018). Estimation of Soil Organic Carbon in Arable Soil in Belgium and Luxembourg with the LUCAS Topsoil Database: Estimation of SOC with the LUCAS Topsoil Database. Eur. J. Soil Sci..

[32] Guerrero C., Wetterlind J., Stenberg B., Mouazen A.M., Gabarrón-Galeote M.A., Ruiz-Sinoga J.D., Zornoza R., Viscarra Rossel R.A. (2015). Do We Really Need Large Spectral Libraries for Local Scale SOC Assessment with NIR Spectroscopy?. Soil Tillage Res..

[33] Jiang Q., Li Q., Wang X., Wu Y., Yang X., Liu F. (2017). Estimation of Soil Organic Carbon and Total Nitrogen in Different Soil Layers Using VNIR Spectroscopy: Effects of Spiking on Model Applicability. Geoderma.

[34] Lacerda M., Demattê J., Sato M., Fongaro C., Gallo B., Souza A. (2016). Tropical Texture Determination by Proximal Sensing Using a Regional Spectral Library and Its Relationship with Soil Classification. Remote Sens..

[35] Liu S., Shen H., Chen S., Zhao X., Biswas A., Jia X., Shi Z., Fang J. (2019). Estimating Forest Soil Organic Carbon Content Using Vis-NIR Spectroscopy: Implications for Large-Scale Soil Carbon Spectroscopic Assessment. Geoderma.

[36] Moura-Bueno J.M., Dalmolin R.S.D., ten Caten A., Dotto A.C., Demattê J.A.M. (2019). Stratification of a Local VIS-NIR-SWIR Spectral Library by Homogeneity Criteria Yields More Accurate Soil Organic Carbon Predictions. Geoderma.

[37] Ogen Y., Zaluda J., Francos N., Goldshleger N., Ben-Dor E. (2019). Cluster-Based Spectral Models for a Robust Assessment of Soil Properties. Geoderma.

[38] Stevens A., Nocita M., Tóth G., Montanarella L., van Wesemael B. (2013). Prediction of Soil Organic Carbon at the European Scale by Visible and near Infrared Reflectance Spectroscopy. PLoS ONE.

[39] Tsakiridis N.L., Tziolas N.V., Theocharis J.B., Zalidis G.C. (2019). A Genetic Algorithm-based Stacking Algorithm for Predicting Soil Organic Matter from Vis–NIR Spectral Data. Eur. J. Soil Sci..

[40] Tsakiridis N.L., Keramaris K.D., Theocharis J.B., Zalidis G.C. (2020). Simultaneous Prediction of Soil Properties from VNIR-SWIR Spectra Using a Localized Multi-Channel 1-D Convolutional Neural Network. Geoderma.

[41] Yang J., Wang X., Wang R., Wang H. (2020). Combination of Convolutional Neural Networks and Recurrent Neural Networks for Predicting Soil Properties Using Vis–NIR Spectroscopy. Geoderma.

[42] Demattê J.A.M., Dotto A.C., Paiva A.F.S., Sato M.V., Dalmolin R.S.D., de Araújo M.d.S.B., da Silva E.B., Nanni M.R., ten Caten A., Noronha N.C. (2019). The Brazilian Soil Spectral Library (BSSL): A General View, Application and Challenges. Geoderma.

[43] Rossel R.V., Behrens T., Ben-Dor E., Brown D.J., Demattê J.A.M., Shepherd K.D., Shi Z., Stenberg B., Stevens A., Adamchuk V. (2016). A Global Spectral Library to Characterize the World’s Soil. Earth-Sci. Rev..

[44] Dangal S., Sanderman J., Wills S., Ramirez-Lopez L. (2019). Accurate and Precise Prediction of Soil Properties from a Large Mid-Infrared Spectral Library. Soil Syst..

[45] Ng W., Minasny B., Montazerolghaem M., Padarian J., Ferguson R., Bailey S., McBratney A.B. (2019). Convolutional Neural Network for Simultaneous Prediction of Several Soil Properties Using Visible/near-Infrared, Mid-Infrared, and Their Combined Spectra. Geoderma.

[46] Sanderman J., Savage K., Dangal S.R.S. (2020). Mid-infrared Spectroscopy for Prediction of Soil Health Indicators in the United States. Soil Sci. Soc. Am. J..

[47] Seybold C.A., Ferguson R., Wysocki D., Bailey S., Anderson J., Nester B., Schoeneberger P., Wills S., Libohova Z., Hoover D. (2019). Application of Mid-Infrared Spectroscopy in Soil Survey. Soil Sci. Soc. Am. J..

[48] Wijewardane N.K., Ge Y., Sanderman J., Ferguson R. (2021). Fine Grinding Is Needed to Maintain the High Accuracy of Mid-infrared Diffuse Reflectance Spectroscopy for Soil Property Estimation. Soil Sci. Soc. Am. J..

[49] Sequeira C.H., Wills S.A., Grunwald S., Ferguson R.R., Benham E.C., West L.T. (2014). Development and Update Process of VNIR-Based Models Built to Predict Soil Organic Carbon. Soil Sci. Soc. Am. J..

[50] Wijewardane N.K., Ge Y., Wills S., Loecke T. (2016). Prediction of Soil Carbon in the Conterminous United States: Visible and near Infrared Reflectance Spectroscopy Analysis of the Rapid Carbon Assessment Project. Soil Sci. Soc. Am. J..

[51] Coutinho M.A., Alari F.D.O., Ferreira M.M., do Amaral L.R. (2019). Influence of Soil Sample Preparation on the Quantification of NPK Content via Spectroscopy. Geoderma.

[52] McDowell M.L., Bruland G.L., Deenik J.L., Grunwald S., Knox N.M. (2012). Soil Total Carbon Analysis in Hawaiian Soils with Visible, near-Infrared and Mid-Infrared Diffuse Reflectance Spectroscopy. Geoderma.

[53] Shao Y., He Y. (2011). Nitrogen, Phosphorus, and Potassium Prediction in Soils, Using Infrared Spectroscopy. Soil Res..

[54] Van Groenigen J.W., Mutters C.S., Horwath W.R., Van Kessel C. (2003). NIR and DRIFT-MIR Spectrometry of Soils for Predicting Soil and Crop Parameters in a Flooded Field. Plant Soil.

[55] Xie H.T., Yang X.M., Drury C.F., Yang J.Y., Zhang X.D. (2011). Predicting Soil Organic Carbon and Total Nitrogen Using Mid- and near-Infrared Spectra for Brookston Clay Loam Soil in Southwestern Ontario, Canada. Can. J. Soil. Sci..

[56] Zhang L., Yang X., Drury C., Chantigny M., Gregorich E., Miller J., Bittman S., Reynolds D., Yang J. (2017). Infrared Spectroscopy Prediction of Organic Carbon and Total Nitrogen in Soil and Particulate Organic Matter from Diverse Canadian Agricultural Regions. CJSS.

[57] Guo Y., Gong P., Amundson R. (2003). Pedodiversity in the United States of America. Geoderma.

[58] Veum K.S., Sudduth K.A., Kremer R.J., Kitchen N.R. (2015). Estimating a Soil Quality Index with VNIR Reflectance Spectroscopy. Soil Sci. Soc. Am. J..

[59] Xia Y., Ugarte C.M., Guan K., Pentrak M., Wander M.M. (2018). Developing Near- and Mid-Infrared Spectroscopy Analysis Methods for Rapid Assessment of Soil Quality in Illinois. Soil Sci. Soc. Am. J..

[60] Soil Survey Staff (2014). Kellogg Soil Survey Laboratory Methods Manual. Soil Survey Investigations Report No 42.

[61] Savitzky A., Golay M.J. (1964). Smoothing and Differentiation of Data by Simplified Least Squares Procedures. Anal. Chem..

[62] Wold S., Sjöström M., Eriksson L. (2001). PLS-Regression: A Basic Tool of Chemometrics. Chemom. Intell. Lab. Syst..

[63] Breiman L. (2001). Random Forests. Mach. Learn..

[64] Quinlan J.R., Adams S. (1992). Learning with Continuous Classes. Proceedings of the AI’92.

[65] Friedman J.H., Roosen C.B. (1995). An Introduction to Multivariate Adaptive Regression Splines. Stat. Methods Med. Res..

[66] Soil Survey Staff (2014). Keys to Soil Taxonomy.

[67] Orgiazzi A., Ballabio C., Panagos P., Jones A., Fernández-Ugalde O. (2018). LUCAS Soil, the Largest Expandable Soil Dataset for Europe: A Review. Eur. J. Soil Sci..

[68] Mevik B.-H. VIP.R: Implementation of VIP (Variable Importance in Projection) for the “pls” Package. http://mevik.net/work/software/VIP.R.

[69] Kuhn M. (2008). Building Predictive Models in *R* Using the Caret Package. J. Stat. Soft..

[70] Clark R.N., Rencz A.N. (1999). Chapter 1: Spectroscopy of Rocks and Minerals, and Principles of Spectroscopy. Remote Sensing for the Earth Sciences.

[71] Bishop J.L., Lane M.D., Dyar M.D., King S.J., Brown A.J., Swayze G.A. (2014). Spectral Properties of Ca-Sulfates: Gypsum, Bassanite, and Anhydrite. Am. Mineral..

[72] Ben-Dor E., Irons J.R., Epema G.F., Rencz A.N., Ryerson R.A. (1999). Soil Reflectance. Manual of Remote Sensing.

[73] Knox N.M., Skidmore A.K., Schlerf M., de Boer W.F., van Wieren S.E., van der Waal C., Prins H.H.T., Slotow R. (2010). Nitrogen Prediction in Grasses: Effect of Bandwidth and Plant Material State on Absorption Feature Selection. Int. J. Remote Sens..

[74] Zhang Y., Li M., Zheng L., Qin Q., Lee W.S. (2019). Spectral Features Extraction for Estimation of Soil Total Nitrogen Content Based on Modified Ant Colony Optimization Algorithm. Geoderma.

